# The airway epithelium: an orchestrator of inflammation, a key structural barrier and a therapeutic target in severe asthma

**DOI:** 10.1183/13993003.01397-2023

**Published:** 2024-04-04

**Authors:** Richard J. Russell, Louis-Philippe Boulet, Christopher E. Brightling, Ian D. Pavord, Celeste Porsbjerg, Del Dorscheid, Asger Sverrild

**Affiliations:** 1 Institute for Lung Health, NIHR Leicester Biomedical Research Centre, University of Leicester, Leicester, UK; 2 Quebec Heart and Lung Institute, Laval University, Quebec, QC, Canada; 3 Respiratory Medicine, NIHR Oxford Biomedical Research Centre, Nuffield Department of Medicine, University of Oxford, Oxford, UK; 4 Department of Respiratory Medicine and Infectious Diseases, Bispebjerg Hospital, Copenhagen University, Copenhagen, Denmark; 5 Centre for Heart Lung Innovation, Department of Medicine, University of British Columbia, Vancouver, BC, Canada

## Abstract

Asthma is a disease of heterogeneous pathology, typically characterised by excessive inflammatory and bronchoconstrictor responses to the environment. The clinical expression of the disease is a consequence of the interaction between environmental factors and host factors over time, including genetic susceptibility, immune dysregulation and airway remodelling. As a critical interface between the host and the environment, the airway epithelium plays an important role in maintaining homeostasis in the face of environmental challenges. Disruption of epithelial integrity is a key factor contributing to multiple processes underlying asthma pathology. In this review, we first discuss the unmet need in asthma management and provide an overview of the structure and function of the airway epithelium. We then focus on key pathophysiological changes that occur in the airway epithelium, including epithelial barrier disruption, immune hyperreactivity, remodelling, mucus hypersecretion and mucus plugging, highlighting how these processes manifest clinically and how they might be targeted by current and novel therapeutics.

## Shareable abstract

**Epithelial dysfunction is a key contributor in asthma pathology. This review considers the clinical manifestations of asthma, their pathophysiological mechanisms and the impact of current and future therapies in the context of airway epithelial disruption.** https://bit.ly/48lEX1a

## Introduction

Asthma is a heterogeneous disease involving diverse underlying pathobiological mechanisms with different clinical manifestations, typically characterised by excessive inflammatory and bronchoconstrictor responses to the environment. Asthma occurs as a result of the interaction between environmental and host factors over time. Environmental triggers associated with the development and exacerbation of asthma are primarily inhaled antigens, viruses and irritants, such as dust mites, moulds, pollens, smoke and other airborne particulates. However, evidence suggests that broader environmental exposures, including food antigens and an altered microbiome, can also contribute [1–3]. The interaction probably begins with genetic susceptibility, particularly in early-onset asthma [4], leading to immune hyperreactivity, airway remodelling and impaired airway function. The resulting clinical manifestations include variable airflow obstruction, wheezing, dyspnoea, cough, phlegm and exacerbations [5]. The airway epithelium serves as an interface between the body and the external environment, and it is the first line of defence against allergens, pathogens and environmental toxins [6, 7]. In addition to its barrier function, the airway epithelium is a key coordinator of immune responses to environmental stimuli.

Alterations to epithelial structure and function have a central role in the development and clinical features of asthma. In the healthy state, the epithelial immune response to environmental exposures and insults is proportional and sufficient. In patients with asthma, epithelial homeostasis is skewed towards impaired innate defence, persistent type 2 (T2) inflammation, barrier disruption and tissue remodelling [7]. Asthma is a variable disease characterised by generally reversible airway obstruction; however, the resulting airway pathology can become irreversible over time [8]. Epithelial function is influenced by genetic predispositions and early-life exposures, which may contribute to the development of T2 inflammation and airway remodelling [7]. It is important to consider the epithelium not in isolation but rather as one component in the complex and dynamic structural, molecular and inflammatory entity that is the asthmatic airway.

In this review, we first discuss the unmet need in asthma management and provide an overview of the structure and function of the airway epithelium. We then focus on key pathophysiological changes that occur in the airway epithelium, including epithelial barrier disruption, immune hyperreactivity, remodelling, mucus hypersecretion and mucus plugging, highlighting how these processes manifest clinically and how they might be targeted by current and novel therapeutics.

## Unmet needs in asthma management

Historically, it was believed that immune cell infiltration into the airway wall and the associated immune cell activation and cytokine release was responsible for the epithelial abnormalities observed in asthma. However, we now know that the airway epithelium and other structural components of the lung respond directly to environmental risk factors for asthma and are key initiators and orchestrators of the inflammatory cascade [7].

Currently, airway wall inflammation and smooth muscle constriction are the primary targets for asthma treatment, whereas the effects of treatments on the airway epithelium have been less well studied. Controller therapies such as inhaled corticosteroids (ICS) improve asthma symptoms in most patients; however, approximately 5–10% of patients with asthma have severe disease, experiencing symptoms and asthma exacerbations despite receiving maximal doses of controller therapy with adequate treatment adherence [5, 9, 10]. Biologics targeting downstream effectors of inflammation (interleukin (IL)-4, IL-5, IL-13 and IgE) are effective for many patients, but not for those without elevated T2 biomarker levels [11–13]. Some patients may have inflammatory pathways or other mechanisms that are not addressed by biologics that affect downstream inflammatory targets, leading to a partial or no response [14]. Tezepelumab, a recently approved biologic targeting the epithelial cytokine thymic stromal lymphopoietin (TSLP), has demonstrated efficacy in patients without elevated T2 biomarker levels [15]. This suggests that biologics targeting upstream epithelial cytokines may have the potential to treat wide-ranging aspects of asthma pathology. However, it remains unclear whether any of these therapies affect the structure and function of the airway epithelium itself.

## Structure and function of the airway epithelium

**FIGURE 1:**
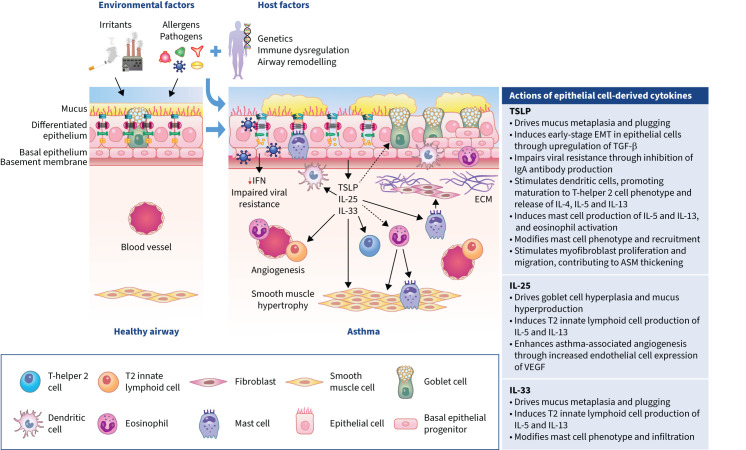
The airway epithelium in healthy individuals and patients with asthma. In the healthy airway, ciliated epithelial cells are connected by tight junctions, forming a physical barrier against infiltration by environmental agents. Goblet cells secrete mucus, which traps pathogens and transports them out of the airways through mucociliary clearance. In asthma, a combination of environmental insults and patient factors leads to epithelial barrier disruption. Epithelial damage stimulates the release of epithelial cytokines (thymic stromal lymphopoietin (TSLP), interleukin (IL)-25 and IL-33), which act on epithelial, subepithelial and immune cell types to drive multiple pathophysiological changes underlying the development, persistence and exacerbation of asthma. These changes include mucus hypersecretion and plugging, impaired viral resistance, airway remodelling, immune hyperreactivity, airway smooth muscle (ASM) hypertrophy and airway hyperresponsiveness. ECM: extracellular matrix; EMT: epithelial-to-mesenchymal transition; IFN: interferon; T2: type 2; TGF: transforming growth factor; VEGF: vascular endothelial growth factor.

The bronchial epithelium is made up of five main cell types, *i.e.* ciliated, club, goblet, basal and suprabasal cells, along with more rare cell types such as ionocytes, neuroendocrine cells and tuft/brush cells in a pseudo-stratified organisation, overlying a basement membrane (figure 1) [16]. Ciliated cells line the airway wall, clearing material trapped in mucus from the cell surface [17]. Club cells secrete substances required for airway protection and repair, including protease inhibitors, proteins that stabilise surfactants, antimicrobial peptides and components of the innate immune response [18–20]. Goblet cells are another secretory cell type, secreting mucins and other glycoproteins that contribute to mucus viscosity [21]. Basal cells include both quiescent stem cells and dividing progenitors, which serve to replenish both secretory and ciliated cells following damage [17]. Data sets obtained using single-cell RNA sequencing technology are now available for the human respiratory tract. These have provided further insights into cellular identities and lineages and have enabled comparative transcriptomic investigations [22].

The physical barrier function of the epithelium is dependent on the integrity of the constituent cells and their adhesions, particularly tight and adherens junctions [6]. The epithelium also serves as an immune barrier, secreting pathogen defence proteins and transporting IgA and IgM from the basolateral membrane to the apical surface, where they bind and trap pathogens in mucus and interact with immune cells [7]. Mucus contains antimicrobial peptides that can neutralise pathogens as they are trapped and removed from the body through mucociliary clearance [19]. Together, the epithelial cells and mucus protect the underlying submucosal layer from air pollutants, allergens and respiratory pathogens [23]. Exposure to air pollutants including particulate matter, such as dust, vehicular pollution, wildfire smoke and cigarette smoke, can disrupt the tight and adherens junctions between epithelial cells. Furthermore, these pollutants induce mucociliary dysfunction characterised by loss of cilia and metaplasia of goblet cells, resulting in increased mucus production. The recognition of particulate matter by epithelial dendritic cells leads to the proliferation of adaptive immune cells and the release of inflammatory cytokines, which induces further epithelial damage and dysfunction [3, 23]. Damage to the epithelium leads to the release of epithelial cytokines, or “alarmins” (TSLP, IL-25 and IL-33), and other cytokines, chemokines and antimicrobial peptides, which recruit and activate innate and adaptive immune cells to mediate necessary and sufficient immune and antiviral responses followed by epithelial repair [24].

**FIGURE 2:**
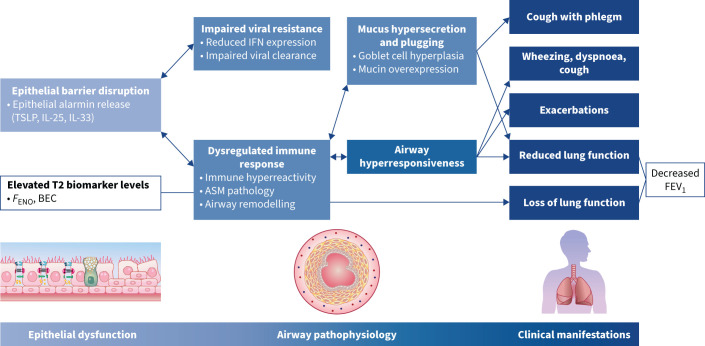
The role of the epithelium in driving clinical manifestations of asthma. Disruption of the airway epithelium triggers the release of epithelial cytokines. Either directly or through downstream inflammatory cascades, epithelial cytokines drive pathophysiological changes to the airways that result in clinical manifestations of asthma, including wheezing, dyspnoea, cough, exacerbations and reversible or permanent loss of lung function. ASM: airway smooth muscle; BEC: blood eosinophil count; *F*_ENO_: fractional exhaled nitric oxide; FEV_1_: forced expiratory volume in 1 s; IFN: interferon; IL: interleukin; T2: type 2; TSLP: thymic stromal lymphopoietin.

Key mechanisms of epithelial dysfunction in the context of common clinical manifestations of asthma (figure 2) are examined in the following sections, along with the underlying pathophysiological mechanisms and potential therapeutic targets. Therapies, either licensed or in development, that target the airway epithelium in severe asthma are summarised in table 1.

**TABLE 1:** Therapeutic drugs/compounds that are available or in development to target the airway epithelium in severe asthma

**Therapy**	**Effect on the airway epithelium and downstream effects**
**Corticosteroids (*e.g.* prednisone, fluticasone, budesonide)**	Restores epithelial cell junctions and barrier integrity [57–59] May inhibit *MUC5AC/5B* expression and goblet cell metaplasia [220] Reduces RBM thickness by reducing deposition of ECM components [172, 173] Reduces airway inflammation [99–102] Reduces the infiltration of immune cells into lung tissue [108, 109]
**Montelukast (LTRA)**	Suppresses cysteinyl leukotriene-induced disruption of tight and adherens junctions in human airway epithelial cells [56]
**Bronchial thermoplasty**	Increases epithelial integrity [63] Reduces ASM mass, collagen deposition and overall RBM thickness [182–184]
**Dupilumab (anti-IL-4/13)**	Reduces levels of nasal albumin, a marker of epithelial barrier dysfunction, in AERD [62] Reduced goblet cell metaplasia in a mouse model of allergic asthma [223] Decreases *F*_ENO_ and serum total IgE levels [113–115]
**Omalizumab (anti-IgE)**	Reduces expression of the high-affinity IgE receptor on plasmacytoid dendritic cells, restoring the antiviral immune response [68–70] Reduces RBM thickness and ECM deposition [188–190] Blocks the interactions of IgE with mast cells and eosinophils, reducing the immune response to allergens [116–119] Reduces serum free IgE concentrations [116]
**Mepolizumab (anti-IL-5)**	Reduces the thickness and density of the ECM component tenascin in the RBM [191] Reduces eosinophil counts in the blood and sputum [120, 121]
**Reslizumab (anti-IL-5)**	Reduces eosinophil counts in the blood and sputum [122–124]
**Benralizumab (anti-IL-5 receptor)**	Decreases ASM mass by depletion of eosinophils in the bronchial lamina propria [192] Reduces eosinophil counts in the blood and sputum [125, 126] Reduces AHR to mannitol [193] Improved ventilation assessed by ^129^Xe MRI in patients with uncontrolled asthma and in those with significant mucus plugging [224]
**Tezepelumab (anti-TSLP)**	Reduces T2 inflammatory biomarker levels [72, 127–130] Reduces AHR to methacholine and mannitol [131, 132, 197] Reduces mucus plugging [13]
**Tozorakimab (anti-IL-33)^#^**	Potential to reduce inflammation and epithelial dysfunction [134]

AERD: aspirin-exacerbated respiratory disease; AHR: airway hyperresponsiveness; ASM: airway smooth muscle; ECM: extracellular matrix; *F*_ENO_: fractional exhaled nitric oxide; IL: interleukin; LTRA: leukotriene receptor antagonist; MRI: magnetic resonance imaging; RBM: reticular basement membrane; T2: type 2; TSLP: thymic stromal lymphopoietin. ^#^: in development.

## Airway epithelial barrier disruption

Airway epithelial barrier disruption allows pathogens to enter the body, driving sensitisation and chronic inflammation; consequently, it is widely regarded as a key driver of asthma initiation, persistence and exacerbation. Many asthma allergens (including those from pollens, insects and fungi) contain active proteases, which can disrupt epithelial tight junctions and increase the infiltration of allergens through the airway wall [25–27]. Allergen activation of mast and T2 innate lymphoid cells promotes the release of mediators such as histamine and IL-13, respectively, which can further disrupt junctional complexes and increase epithelial permeability [28, 29].

The expression of E-cadherin, an adherens junction protein, is reduced in the airway epithelium of people with asthma. This may occur as a result of epithelial-to-mesenchymal transition, in which epithelial cells lose their polarity, adhesiveness and anchorage to the basal membrane and acquire mesenchymal features, such as enhanced migratory abilities [30–32]. Dendritic cells receive tonic inhibitory signals from E-cadherin-mediated association with epithelial cells. Loss of inhibition in asthma could activate dendritic cells, leading to allergic sensitisation [33, 34]. Epithelial damage triggers the release of fibrogenic cytokines such as IL-1β, interferon (IFN)-γ, tumour necrosis factor (TNF), IL-4 and IL-13, which can themselves decrease the expression of junctional proteins, creating a cycle of epithelial damage, airway remodelling and inflammation [7]. *In vitro* studies suggest that TSLP may play a key role in this cycle. Loss of E-cadherin can enhance the production of TSLP, which can induce epithelial-to-mesenchymal transition in epithelial cells *via* upregulation of transforming growth factor-β [35, 36]. Genome-wide association studies have also identified several key epithelial-expressed genes and gene networks associated with an increased asthma risk, including *TSLP*, *IL33* and *IL1RL1* (an IL-33 receptor) [37]. It should be noted, however, that direct evidence for the effect of these factors on epithelial integrity is lacking.

Epithelial repair mechanisms appear to be dysregulated in asthma, exacerbating impaired epithelial integrity. An increase in poorly differentiated basal cells has been observed in addition to reduced proliferation of epithelial cells and defects in wound healing [38–40]. Pre-school children with severe wheezing have shown impaired epithelial proliferation and wound healing following viral infection, which may contribute to airway remodelling and asthma pathogenesis [41].

### Impaired viral resistance

Viral infection of the airways also has profound effects on epithelial barrier function [42]. Facilitated by a disrupted barrier, viral infection activates epithelial cells, triggering innate immune and T2 inflammatory responses, and amplifying the overall inflammation commonly present in asthma [42–44]. However, the immune response to viral infection appears to be deficient and delayed in people with asthma, with reduced production of type I IFN and other cytokines in epithelial and dendritic cells leading to greater damage to the airway epithelium [45–47]. An asthma-associated long form of TSLP is produced by epithelial cells following viral infection. It inhibits the production of IgA antibodies, which are believed to neutralise viruses at mucosal surfaces [48]. Viral infection also disrupts junctional complexes and inhibits wound repair, further reducing barrier integrity and driving the cycle of epithelial damage [49–51]. The release of basic fibroblast growth factor and matrix metalloproteinase by virus-infected epithelial cells leads to fibroblastic repair, including increased cell death and extracellular matrix (ECM) deposition [42].

### Clinical manifestations and therapeutics

Over time, environmental interactions with an impaired epithelial barrier and other host factors lead to disordered airway physiology, ultimately producing the characteristic symptoms of asthma. Currently, there are no clinically relevant indicators of epithelial disruption itself and there are limited clinical data on the impact of currently available therapies on epithelial barrier repair.

Long-acting β-agonists may have a protective effect on epithelial barrier integrity [52, 53] and reduce inflammatory signals generated by airway epithelial cells [54, 55]. Montelukast has been shown to suppress cysteinyl leukotriene-induced disruption of tight and adherens junctions in human airway epithelial cells [56]. *In vitro* studies suggest that the corticosteroids dexamethasone and budesonide can restore epithelial cell junctions and barrier integrity [57–59]. However, corticosteroid treatment can elicit time- and concentration-dependent epithelial cell death, resulting in barrier dysfunction [60, 61]. This may underlie some aspects of corticosteroid resistance, in which symptoms that are not responsive to ICS or oral corticosteroids arise from excessive corticosteroid exposure and resulting injury to the epithelium. The effects of biologics on airway epithelial barrier dysfunction are not yet known. However, dupilumab, a monoclonal antibody that blocks IL-4 and IL-13 signalling by targeting IL-4 receptor α, was shown to reduce levels of nasal albumin, a marker of epithelial barrier dysfunction, in aspirin-exacerbated respiratory disease [62]. Bronchial thermoplasty has been shown to increase epithelial integrity in bronchial biopsies, and *in vitro* data have shown that exposure to thermoplasty-equivalent temperatures results in an initial significant decrease in the number of viable epithelial cells, followed by a recovery over the subsequent 14 days [63]. Although this finding demonstrates an epithelial response to thermal injury, the functional properties of the repaired epithelium are not known. Additionally, this small study did not demonstrate a relationship between improvements in epithelial integrity and asthma control.

IFN-α/β expression in bronchial epithelial cells and subepithelial monocytes and macrophages during rhinovirus infection was found to be deficient in patients with atopic asthma, which in turn was associated with adverse clinical outcomes [64]. In contrast, increased IFN expression by subepithelial cells during infection was also related to a greater viral load and severity of disease. Although a trial of inhaled IFN-β therapy failed to meet its primary end-point, restoration of IFN levels is a potential therapeutic approach for controlling virally induced asthma exacerbations [65]. In children with allergic asthma, omalizumab (an anti-IgE therapy) reduced the duration and frequency of rhinovirus infections and decreased the rate and severity of rhinovirus-induced exacerbations [66–68]. Omalizumab has also been shown to reduce expression of the high-affinity IgE receptor on plasmacytoid dendritic cells compared with placebo in patients with atopic and non-atopic asthma, which is thought to restore antiviral immune responses [68–70]. Consistent with enhanced antiviral resistance, allergen immunotherapy increased IFN and IL-6 expression in response to a viral mimic in patients with allergic asthma triggered by house dust mites [71]. A study has shown that blocking TSLP stabilises the bronchial immune response to viral stimulation. Tezepelumab had no effect on IFN-β and IFN-λ release or the total viral load in bronchial cells in response to viral challenge [72]. Conversely, ICS have been shown to suppress pro-inflammatory (IL-6 and IL-8) and antiviral (IFN-λ1) cytokine production and increase viral replication [73].

## Immune hyperreactivity

In asthma, immune hyperreactivity is the exaggerated immune response of the airways to an environmental trigger. This results in excessive production and release of pro-inflammatory cytokines, driving key components of asthma including airway inflammation and airway hyperresponsiveness (AHR). Exposure to inhaled asthma triggers causes the release of epithelial cytokines (TSLP, IL-25 and IL-33), which promotes both innate and adaptive T2 immune responses.

In patients with asthma, the epithelium produces higher levels of cytokines, both in the steady state and following challenges by inhaled allergens and viral infection [74]. Elevated levels of TSLP, IL-25, IL-33 and the IL-33 receptor serum stimulation-2 (ST2) in the serum and bronchial tissue of patients with asthma have also been found to correlate with T2 inflammation and disease severity [74]. Elevated serum and sputum levels of IL-25 are associated with allergic asthma, and elevated serum levels of IL-33 are associated with allergic and eosinophilic asthma [75–77].

IL-25 and IL-33 (released by tuft cells and basal cells, respectively) activate T2 innate lymphoid cells to produce IL-5 and IL-13, promoting T2 inflammation [7]. TSLP produced by basal cells stimulates dendritic cells, which then activate T-helper 2 cells to release the T2 cytokines IL-4, IL-5 and IL-13 [78]. TSLP is also a potent activator of mast cells, triggering the release of T2 cytokines including IL-5 and IL-13 [79]. Eosinophils activated by IL-5 release cysteinyl leukotrienes, which are potent bronchoconstrictors [80, 81]. IL-4 and IL-13 stimulate B-cells to produce IgE, which causes AHR and mast cell degranulation, thereby increasing vascular permeability [82]. IL-4 and IL-13 also affect Toll-like receptor expression, potentially increasing viral responses [83]. IL-13 stimulates goblet cell proliferation, leading to increased mucus production [84]. Eosinophils can induce airway remodelling *via* their effects on airway smooth muscle (ASM), mast cells and epithelial cells [81, 85]. Mast cell infiltration into ASM is also involved in T2-independent asthma pathology [86, 87].

Symptoms of asthma also occur in patients without significant T2 inflammation. Non-T2 asthma is thought to encompass a range of endotypes, including paucigranulocytic and neutrophilic subtypes [88]. Neutrophilic inflammation can be caused by smoking, COPD, viral infection and obesity, and is driven by type 1 and T-helper 17 cytokines, including IFN-γ, TNF-α, IL-8 and IL-17 [89–91]. Neutrophil recruitment and autophagy can activate eosinophils and mast cells, contributing to epithelial damage and the disruption of tight junctions [92]. However, there is some uncertainty regarding the existence and definition of a specific neutrophilic asthma endotype. Raised neutrophil levels can arise from treatment with high doses of inhaled glucocorticosteroids [93]. Furthermore, studies have shown a lack of improvement in asthma outcomes when key pathways of neutrophil recruitment are interrupted [94].

### Clinical manifestations and therapeutics

Chronic T2 inflammation triggers symptoms of acute bronchoconstriction including cough, chest tightness, dyspnoea and wheezing, leading to exacerbations [7].

T2 airway inflammation driven by mast cells and eosinophils is reflected in elevated blood and sputum eosinophil counts and fractional exhaled nitric oxide (*F*_ENO_) levels [95, 96]. Nitric oxide is produced primarily in the bronchial epithelium by inducible nitric oxide synthase. Increased expression of T2 cytokines such as IL-4 and IL-13 results in overexpression of inducible nitric oxide synthase and increased *F*_ENO_ levels, which further promotes T2 inflammation [96, 97]. Chronic inflammation and frequent exacerbations can lead to a reduction in lung function, typically assessed as a decrease in the forced expiratory volume in 1 s (FEV_1_) [98]. Several genes expressed in the epithelium, including *TSLP*, *IL13* and *TGFB1*, have been associated with decreased FEV_1_.

Current standard-of-care therapies targeting immune hyperreactivity are primarily ICS, which are usually given in combination with a long-acting bronchodilator. Corticosteroids reduce airway inflammation by promoting anti-inflammatory gene expression [99, 100] and repressing pro-inflammatory gene expression [99, 101, 102]. This reduces the survival, activation and/or migration of eosinophils, lymphocytes, macrophages and dendritic cells [103–107]. Corticosteroids also reduce the infiltration of immune cells into lung tissue by decreasing the expression of adhesion proteins and secretion of chemokines (table 1) [108, 109]. Overall, these treatments manage symptoms associated with airway inflammation without altering the underlying disease processes. However, they may not be sufficient to achieve asthma control in all patients [110–112].

Epithelial damage triggers the release of epithelial cytokines that initiate T2 inflammatory cascades. Biologic therapies targeting downstream mediators of T2 inflammation have provided significant improvements in outcomes for some patients with severe asthma. By blocking the action of cytokines such as IL-4, IL-5 and IL-13, biologics can help to reduce T2 inflammation and associated severe asthma symptoms, including exacerbations. Compared with placebo, dupilumab decreased *F*_ENO_ and serum total IgE levels, reduced the annualised asthma exacerbation rate and increased pre-bronchodilator FEV_1_ (table 1) [113–115]. Omalizumab has been shown to block the interactions of IgE with mast cells and eosinophils, reducing the immune response to allergens [116–119]. Omalizumab also reduces serum free IgE concentrations and exacerbations compared with placebo [116], but has little effect on blood eosinophil counts (BECs) or *F*_ENO_ levels [117, 118]. Three biologics targeting IL-5 signalling (mepolizumab [120, 121], reslizumab [122–124] and benralizumab [125, 126]) reduced eosinophil counts in the blood and sputum, leading to reduced exacerbations compared with placebo, but without effects on serum IgE levels, *F*_ENO_ levels or AHR.

Biologics targeting epithelial cytokines upstream of T2 immune responses (TSLP and IL-33) may provide broader physiological effects. Tezepelumab (an anti-TSLP therapy) reduced levels of airway epithelial IL-33 and T2 inflammatory cytokines (blood eosinophils, airway eosinophils, *F*_ENO_, IL-5, IL-13 and periostin), reduced exacerbations and improved FEV_1_ compared with placebo [72, 127–130]. Additionally, tezepelumab was effective in patients with low baseline BECs and *F*_ENO_ levels (although less so than in patients with higher baseline levels of these biomarkers) [15, 130]. Tezepelumab also reduced AHR to mannitol, indicating that TSLP blockade might have additional benefits in asthma beyond reducing T2 airway inflammation [131, 132]. Studies on anti-IL-33 monoclonal antibodies are underway [133–135].

## Airway remodelling

### ASM pathology and AHR

AHR refers to exaggerated bronchoconstriction in response to an inhaled irritant; it has variable components attributed to inflammatory responses and persistent components related to structural changes to the airway [136]. In asthma, epithelial cytokines have been shown to act on myofibroblasts and ASM cells, leading to changes in the ASM and subsequent AHR.

Structural changes observed in asthmatic airways include ASM cell hypertrophy, hyperplasia and hypercontractility [137]. Early ASM pathology may be more strongly associated with asthma development than eosinophilia or subepithelial ECM deposition [138, 139]. AHR to inhaled mannitol is associated with (primarily chymase-positive) mast cell infiltration of the epithelium and/or ASM [140], depending on coexisting T2 inflammation [87]. TSLP and IL-33 increase carboxypeptidase A3 expression in mast cell precursors, causing a phenotypic switch associated with AHR [141]. TSLP expression is also associated with chymase-positive mast cell infiltration of the airways in patients with asthma [140]. Upon stimulation by TSLP, mast cells and ASM cells produce a range of chemokines and cytokines that contribute to ASM pathology and AHR, including TSLP, IL-13 and IL-33 [86, 142–144]. ASM thickening is probably due to a combination of myofibroblast proliferation and migration of myofibroblasts and ASM cells, both of which could be stimulated by TSLP and/or other epithelial factors [145, 146]. Increased expression of ECM components (collagen III and laminin) within the ASM layer has been shown to correlate with airway function (FEV_1_ reversibility) in patients with asthma [147].

Angiogenesis is also a feature of airway remodelling in asthma, driven primarily by vascular endothelial growth factor (VEGF). Epithelial cytokines, in particular IL-25, have been implicated in asthma-associated angiogenesis, increasing the proliferation of endothelial cells at least in part by increasing VEGF/VEGF receptor expression [148, 149].

### ECM deposition and subepithelial fibrosis

Imbalances in the disposition and degradation of subepithelial ECM can lead to subepithelial fibrosis, airway wall thickening and airflow obstruction [150, 151]. Fibrosis is related to increased deposition of collagen I, III and V, as well as other ECM components such as fibronectin and tenasin [152]. Increased ECM production by epithelial cells and fibroblasts can be stimulated by inflammatory factors released by eosinophils and other immune cells upon epithelial disruption [153–158]. For example, TSLP can activate lung fibroblasts *via* stimulation of lung macrophages and mast cells [159–162]. However, remodelling has been observed in biopsies from pre-school children without associated elevated eosinophil cell counts [111, 112, 138, 139]. Low-grade airway remodelling can also be observed in patients who are atopic and without asthma, but remodelling increases with the development of asthma [163]. It has also been suggested that bronchoconstriction can induce fibrotic airway remodelling independently of allergen exposure and eosinophilia, contributing to the cycle of injury and defective repair observed in asthma [164]. Finally, mechanostimulation during bronchospasms can activate epithelial cells to produce transforming growth factor-β, endothelin and granulocyte–macrophage colony-stimulating factor, recruiting dendritic and other immune cells to further potentiate immune hyperresponsiveness and promote fibrotic protein synthesis in fibroblasts [165, 166].

### Clinical manifestations and therapeutics

Increased ASM mass in asthmatic airways contributes to airway obstruction, bronchospasms and AHR, ultimately leading to loss of lung function [167]. Fibrotic airway remodelling is a long-term and potentially irreversible modification of airway structure and is a major contributor to AHR and loss of lung function [8]. Over time, remodelling causes airways to become stiff, thickened and narrowed. Clinically, this often presents as variable airway obstruction or airflow limitation, typically recorded as a decrease in FEV_1_ in response to a challenge agent, and other symptoms including cough, wheezing, dyspnoea and increased exacerbation risk [8, 136, 168, 169]. AHR can present without respiratory symptoms in individuals who are atopic and it may represent an intermediate state in the development of asthma [170].

Inhaled bronchodilators such as long-acting β-agonists decrease ASM contractility to improve airflow, which is also an effect of ICS secondary to their anti-inflammatory action [171]. Although some studies have shown that treatment with high-dose ICS reduces deposition of certain ECM components such as collagen and tenascin [172, 173], results have been contradictory and dependent on doses that limit their clinical application [174–181]. Bronchial thermoplasty is the only licensed treatment to consistently show reductions in ASM mass in patients with asthma and has been shown to reduce collagen deposition and overall reticular basement membrane (RBM) thickness (table 1) [182–184]. However, the mechanisms underpinning thermoplasty have yet to be elucidated and it remains unclear whether a reduction in ASM mass is sufficient to improve asthma control [63, 185, 186].

Omalizumab improved AHR in response to acetylcholine challenge in one study of patients with allergic asthma, but not in two other studies investigating responses to methacholine or AMP [119, 136, 187]. Omalizumab has been shown to reduce RBM thickness, potentially also reducing ECM deposition by decreasing mesenchymal cell proliferation (table 1) [188–190]. In a study of bronchial biopsies from patients with mild atopic asthma, mepolizumab reduced the thickness and density of the ECM component tenascin in the RBM [191]. Biopsies from patients with eosinophilic asthma found that benralizumab decreased ASM mass compared with placebo, a finding attributed to the depletion of eosinophils in the bronchial lamina propria [192]. Benralizumab also demonstrated improvements in AHR to mannitol over 12 weeks in an open-label study of patients with eosinophilic asthma [193].

The epithelial cytokines TSLP and IL-33 may be promising therapeutic targets for reducing airway remodelling. Blocking TSLP has been shown to inhibit airway remodelling in animal models [194–196], and blocking IL-33 blunted persistent inflammation and remodelling and prevented exacerbations in a model of chronic airway inflammation [135]. Tezepelumab is the only biologic that has been shown in placebo-controlled trials to consistently improve AHR to methacholine and mannitol in a broad population of patients with severe asthma; this may occur through modification of mast cell phenotype and infiltration [131, 132, 197]. However, studies for other biologics have not yet assessed this outcome and the clinical relevance of improved AHR in terms of other asthma outcomes remains unclear.

## Mucus hypersecretion and mucus plugging

In the healthy airway, mucus provides a layer of defence against pathogens, dust and other inhaled irritants, by trapping them within a thick hydrogel. The underlying ciliated cells propel the mucus towards the oral cavity, preventing all but nanoscale materials from diffusing through the epithelial barrier [7]. Macromolecules called mucins provide the viscous, sticky properties of mucus. In asthmatic airways, both hypersecretion and alterations in the composition of the mucus are often observed. Mucin content has been found to be increased by up to 15% in obstructive conditions [198]. Evidence from patients with asthma and mouse models suggests that the release of IL-13 upregulates mucin gene expression in goblet cells. Specifically, *MUC5AC* is upregulated in people with asthma, particularly in those with the eosinophilic T2 endotype [199–203]. *MUC5AC* is also a susceptibility gene for moderate-to-severe asthma [204]. Mucus hypersecretion can also occur in non-T2 asthma, and mouse studies have shown that neutrophilic inflammation is associated with increased expression of *CLCA1*, *MUC4*, *MUC5AC* and *MUC5B* [205]. Goblet cell hyperplasia, metaplasia and aberrant spread to the peripheral airways are also features of the asthmatic epithelium, with animal studies suggesting this may result from increased expression of IL-4, IL-9 and IL-13 [206–211]. Prolonged exposure to IL-13 also appears to disrupt the development and function of ciliated epithelial cells, affecting their ability to clear mucus from the airway [212]. As upstream mediators of IL-5 and IL-13, IL-33 and TSLP have been implicated as drivers of mucus metaplasia and plugging [213, 214]. Chronic bronchoconstriction also induces goblet cell proliferation, basement membrane thickening and mucus secretion, which worsen airway obstruction [164].

### Clinical manifestations and therapeutics

Goblet cell hyperplasia and mucus hypersecretion can present clinically as increased airflow obstruction, cough with phlegm and exacerbations, and poorer asthma control [215]. Hyperproduction and failed clearance of highly viscous mucus can lead to the formation of mucus plugs. Patients with mucus plugs exhibit marked eosinophilia and increased expression of T2 cytokines [216, 217]. Mucus plugging is correlated with the severity of airflow obstruction and lung function decline (decreased FEV_1_), and has been observed in the airways of patients with fatal asthma [216–218]. However, the volume of expectorated sputum does not reflect distal mucus hypersecretion. Mucus hypersecretion in the small airways can lead to clinically significant mucus plugging but only small volumes of expectorated sputum, whereas mucus hypersecretion in the large airways may cause little or no mucus plugging but large volumes of expectorated sputum [219].

Pre-clinical studies suggest that corticosteroids inhibit *MUC5AC*/*5B* expression and goblet cell metaplasia (table 1) [220]. However, longitudinal studies suggest that mucus plugs can persist despite the use of bronchodilators and ICS/oral corticosteroids [216, 217]. A long-acting muscarinic antagonist has been shown to inhibit IL-13-induced goblet cell metaplasia in human airway epithelial cells, which may protect against mucus hypersecretion [221]. Long-acting muscarinic antagonists also promoted cilia-driven flow and ciliary activity in mouse and human airway epithelium, supporting the removal of pathogens through enhanced mucociliary clearance [222]. Dupilumab also reduced goblet cell metaplasia in a mouse model of allergic asthma [223]. A study showed that a single dose of benralizumab improved ventilation assessed by ^129^Xe magnetic resonance imaging in patients with uncontrolled asthma and in those with significant mucus plugging [224]. Tezepelumab is the only biologic that has been shown to reduce mucus plugging in a placebo-controlled trial, which correlated with improvements in BECs, *F*_ENO_ levels and FEV_1_, and reductions in IL-5 and IL-13 levels [214].

## Conclusion

The evidence presented here indicates that the airway epithelium is an important initiator of inflammatory responses to environmental triggers and makes complex contributions to asthma pathophysiology. Research increasingly points to epithelial disruption as an early event in asthma pathogenesis, but there is currently no way to clinically detect early changes in the airway epithelium. Biologics targeting epithelial cytokines have greatly improved the control of severe asthma symptoms by inhibiting excessive inflammatory responses, but their potential effects on epithelial barrier function are not well understood. Further research is needed to identify biomarkers of early epithelial dysfunction, as well as additional mechanisms and therapeutic targets that promote epithelial repair and homeostasis. This will enable early, targeted intervention to break the injury–inflammation cycle underlying asthma development and alter disease progression. It will also be important to better elucidate non-T2 pathways in asthma and potential immune interactions with the airway epithelium.

## Shareable PDF

10.1183/13993003.01397-2023.Shareable1 This one-page PDF can be shared freely online.
Shareable PDF ERJ-01397-2023.Shareable
