# Supplementary material for: The airway epithelium: an orchestrator of inflammation, a key structural barrier and a therapeutic target in severe asthma
Source: Eur Respir J. 2024 Apr 4;63(4):2301397. doi: 10.1183/13993003.01397-2023 (PMC10991852; doi:10.1183/13993003.01397-2023)
Supplement: Supplementary file 1 [file ERJ-01397-2023.Shareable.pdf]

EUROPEAN RESPIRATORY JOURNAL
REVIEW
R.J. RUSSELL ET AL.

SHAREABLE PDF

# The airway epithelium: an orchestrator of inflammation, a key structural barrier and a therapeutic target in severe asthma

Richard J. Russell[1], Louis-Philippe Boulet[2], Christopher E. Brightling[1], Ian D. Pavord[3], Celeste Porsbjerg[4], Del Dorscheid[5] and Asger Sverrild[4]

[1]Institute for Lung Health, NIHR Leicester Biomedical Research Centre, University of Leicester, Leicester, UK. [2]Quebec Heart and Lung Institute, Laval University, Quebec, QC, Canada. [3]Respiratory Medicine, NIHR Oxford Biomedical Research Centre, Nuffield Department of Medicine, University of Oxford, Oxford, UK. [4]Department of Respiratory Medicine and Infectious Diseases, Bispebjerg Hospital, Copenhagen University, Copenhagen, Denmark. [5]Centre for Heart Lung Innovation, Department of Medicine, University of British Columbia, Vancouver, BC, Canada.

Corresponding author: Richard J. Russell (rjr22@leicester.ac.uk)

Check for updates

Shareable abstract (@ERSpublications)
**Epithelial dysfunction is a key contributor in asthma pathology. This review considers the clinical manifestations of asthma, their pathophysiological mechanisms and the impact of current and future therapies in the context of airway epithelial disruption.** https://bit.ly/48lEX1a

**Cite this article as:** Russell RJ, Boulet L-P, Brightling CE, *et al*. The airway epithelium: an orchestrator of inflammation, a key structural barrier and a therapeutic target in severe asthma. *Eur Respir J* 2024; 63: 2301397 [DOI: 10.1183/13993003.01397-2023].

This extracted version can be shared freely online.

Copyright ©The authors 2024.

This version is distributed under the terms of the Creative Commons Attribution Licence 4.0.

Received: 18 Aug 2023
Accepted: 15 Feb 2024

## Abstract

Asthma is a disease of heterogeneous pathology, typically characterised by excessive inflammatory and bronchoconstrictor responses to the environment. The clinical expression of the disease is a consequence of the interaction between environmental factors and host factors over time, including genetic susceptibility, immune dysregulation and airway remodelling. As a critical interface between the host and the environment, the airway epithelium plays an important role in maintaining homeostasis in the face of environmental challenges. Disruption of epithelial integrity is a key factor contributing to multiple processes underlying asthma pathology. In this review, we first discuss the unmet need in asthma management and provide an overview of the structure and function of the airway epithelium. We then focus on key pathophysiological changes that occur in the airway epithelium, including epithelial barrier disruption, immune hyperreactivity, remodelling, mucus hypersecretion and mucus plugging, highlighting how these processes manifest clinically and how they might be targeted by current and novel therapeutics.
